# The impact of the introduction of critical care outreach services in England: a multicentre interrupted time-series analysis

**DOI:** 10.1186/cc6163

**Published:** 2007-09-18

**Authors:** Haiyan Gao, David A Harrison, Gareth J Parry, Kathleen Daly, Christian P Subbe, Kathy Rowan

**Affiliations:** 1 Intensive Care National Audit & Research Centre (ICNARC), Tavistock House, Tavistock Square, London WC1H 9HR, UK; 2 National Institute of Clinical Outcomes Research, University College London, Suite 501, Heart Hospital, Westmoreland Street, London W1G 8PH, UK; 3 Children's Hospital Boston, 300 Longwood Avenue, Boston, MA 02115, USA; 4 Intensive Care Unit, St Thomas' Hospital, Lambeth Palace Road, London SE1 7EH, UK; 5 Wrexham Maelor Hospital, Croesnewydd Road, Wrexham LL13, UK

## Abstract

**Introduction:**

Critical care outreach services (CCOS) have been widely introduced in England with little rigorous evaluation. We undertook a multicentre interrupted time-series analysis of the impact of CCOS, as characterised by the case mix, outcome and activity of admissions to adult, general critical care units in England.

**Methods:**

Data from the Case Mix Programme Database (CMPD) were linked with the results of a survey on the evolution of CCOS in England. Over 350,000 admissions to 172 units between 1996 and 2004 were extracted from the CMPD. The start date of CCOS, activities performed, coverage and staffing were identified from survey data and other sources. Individual patient-level data in the CMPD were collapsed into a monthly time series for each unit (panel data). Population-averaged panel-data models were fitted using a generalised estimating equation approach. Various potential outcomes reflecting possible objectives of the CCOS were investigated in three subgroups of admissions: all admissions to the unit, admissions from the ward, and unit survivors discharged to the ward. The primary comparison was between periods when a formal CCOS was and was not present. Secondary analyses considered specific CCOS activities, coverage and staffing.

**Results:**

In all, 108 units were included in the analysis, of which 79 had formal CCOS starting between 1996 and 2004. For admissions from the ward, CCOS were associated with significant decreases in the proportion of admissions receiving cardiopulmonary resuscitation before admission (odds ratio 0.84, 95% confidence interval 0.73 to 0.96), admission out of hours (odds ratio 0.91, 0.84 to 0.97) and mean Intensive Care National Audit & Research Centre physiology score (decrease in mean 1.22, 0.31 to 2.12). There was no significant change in unit mortality (odds ratio 0.97, 0.87 to 1.08) and no significant, sustained effects on outcomes for unit survivors discharged alive to the ward.

**Conclusion:**

The observational nature of the study limits its ability to infer causality. Although associations were observed with characteristics of patients admitted to critical care units, there was no clear evidence that CCOS have a big impact on the outcomes of these patients, or for characteristics of what should form the optimal CCOS.

## Introduction

Critical care outreach services (CCOS) were introduced widely into the National Health Service (NHS) in England in 2000 as an important component of the vision for the future of critical care services [1]. The three main objectives of CCOS were to avert admissions or ensure timely admission to critical care, to enable discharges from critical care, and to share skills with ward staff. There was no prescribed model for CCOS; Critical Care Networks and NHS Trust Critical Care Delivery Groups were encouraged to develop their own locally customised service. Despite little evidence for their benefit, CCOS were introduced without any formal prospective evaluation.

A wide range of services falling under the umbrella of CCOS have been developed, introduced, incrementally implemented and improved over time [2]. These services vary in terms of their objectives (such as meeting one or more of the three main objectives or other additional objectives), activities (such as direct bedside support, follow-up of patients discharged from critical care to the ward, or education and training), staffing (such as doctor-led or nurse-led, or size of team), hours of work (such as round the clock or office hours) and coverage of wards (such as selected wards only or complete coverage) [3]. A systematic review on the effectiveness of CCOS [4] indicated that published research on the impact of CCOS is limited, there is insufficient evidence to confirm their effectiveness, and more comprehensive research is needed. As a result of the wide variation in the models of service delivery adopted and potentially wide variation in the stage of implementation and use, CCOS cannot now be evaluated using the gold-standard research design, a multicentre, randomised controlled trial.

The aim of this study was to undertake a multicentre, interrupted time-series analysis of the impact of CCOS at the critical care unit level, as characterised by the case mix, outcome and activity of admissions to adult, general critical care units participating in the Case Mix Programme, which is the national comparative audit of critical care in England, Wales and Northern Ireland.

## Materials and methods

The analysis sought to examine trends in pre-specified outcomes over time in those critical care units participating in the Case Mix Programme for which CCOS data were available from a previously completed survey.

### Data sources

#### Case Mix Programme Database

The Case Mix Programme Database (CMPD) is a high-quality clinical database of case mix, outcome and activity data on consecutive admissions to adult, general critical care units in England, Wales and Northern Ireland [5]. Data are collected by trained data collectors according to precise rules and definitions, and are validated both locally and centrally before being pooled into the CMPD. A total of 393,205 validated admissions to 172 critical care units between January 1996 and December 2004 were extracted from the CMPD.

The Intensive Care National Audit & Research Centre (ICNARC) physiology score is an illness severity score calculated from the ICNARC risk prediction model [6], based on physiological measurements from the 24 hours after admission to critical care. Admissions were classified as either medical, elective surgical, or emergency surgical, on the basis of the source of admission to the unit and the National Confidential Enquiry into Perioperative Death classification of surgery, as described previously [5].

### Survey data and other sources

The results of a national survey of the evolution of CCOS in England [3] were used to identify units with formal CCOS, to characterise the CCOS in terms of the activities undertaken, coverage and staffing, and to identify important time-dependent confounders.

A total of 191 acute NHS hospitals in England completed the survey. The survey data were validated extensively by a software data entry check, random-sample double data entry, and data cleaning.

The following time-dependent variables were identified from the survey and, where necessary, other sources.

The primary comparison was between periods when a formal CCOS was and was not present in the hospital housing the critical care unit, defined as at least one member of staff with funded time dedicated to the CCOS. Hospitals that were represented both in the CMPD and in survey data were contacted for details of the date on which the CCOS formally started, because this was not included in the survey.

Secondary comparisons were performed by using the following variables to characterise each CCOS:

1. Aspects of outreach activity, eight binary variables: (a) ward follow-up, (b) outpatient follow-up, (c) telephone advice, (d) direct bedside clinical support, (e) informal bedside teaching, (f) formal educational courses, (g) use of physiological track and trigger warning systems, and (h) audit and evaluation of outreach activity.

2. Coverage of CCOS, two categorical variables: (a) temporal (24 hours and 7 days a week; 12 to 23 hours and 7 days per week; less than 12 hours and 7 days per week; selected days), and (b) locational (all wards/selected wards only).

3. Staffing of CCOS, two categorical variables: (a) no medical involvement or some medical involvement (medical staff with dedicated funded sessions allocated to the CCOS), and (b) small team (fewer than three whole-time equivalent staff per ten level 3 or flexible level 2/3 beds) or large team (three or more whole-time equivalent staff per ten level 3 or flexible level 2/3 beds).

All analyses were adjusted for the following confounding variables: number of level 3 beds (general and specialist); number of level 2 beds (general and specialist); number of flexible level 2/3 beds (general and specialist); presence of a standalone general high-dependency unit; teaching status; Foundation Trust status; tertiary referral centre; presence of a 'hospital at night' service; presence of an acute pain team; presence of a nutrition team; availability of non-invasive ventilation on general wards; presence of an overnight ventilation facility in theatre/recovery; use of the Acute Life-threatening Events Recognition and Treatment (ALERT) course, or similar, for ward staff; presence of a formal resuscitation policy.

The timings of the opening of standalone general high-dependency units, granting of Foundation Trust status and initiation of 'hospital at night' services were not included in the survey. These were sought from individual hospitals or from the Department of Health or Modernisation Agency websites.

### Outcome measures

A variety of potential outcomes that might reflect the CCOS objectives of averting admissions, ensuring timely admission and enabling discharge were investigated in the following three subgroups of admissions.

1. All admissions to the unit: proportion of admissions direct from the ward.

2. Admissions from a ward in the same hospital: (a) proportion of admissions receiving cardiopulmonary resuscitation (CPR) during 24 hours before admission, (b) proportion of admissions out of hours (22:00 to 06:59), (c) mean and SD of the ICNARC physiology score, (d) proportion of admissions having all active treatment withdrawn, and (e) mortality in the unit.

3. Unit survivors discharged to the ward: (a) proportion of discharges occurring out of hours (22:00 to 06:59), (b) proportion of discharges designated as an 'early discharge due to shortage of beds', (c) hospital mortality, and (d) proportion of patients readmitted to the unit within 48 hours of discharge.

### Statistical analyses

The interrupted time-series analysis included all admissions in the CMPD from critical care units located in hospitals for which a completed survey form was received. Units were excluded if we were unable to ascertain the formal start date for the CCOS. Missing data in the time-dependent variables identified from the survey were replaced with the last value carried forward unless all values from 1996 to 2004 were missing, in which case that unit was excluded.

Time series consist of sets of values for the same variables collected at regular or irregular intervals. Data in the CMPD are collected on an individual patient basis; however, collapsing the data into a time series of monthly average values for each critical care unit enabled us to use statistical techniques to model trends and cycles over time. Population-averaged panel-data models were fitted by using a generalised estimating equation approach, with robust (Huber–White) variance–covariance estimates to account for clustering at the unit level [7], and an autoregressive correlation structure of order 1 within units over time.

The primary analysis was on the presence of a formal CCOS. Lagged effects over two months were included in the model because the effects of introducing a new service are not likely to be evident immediately after the introduction. Secondary analyses were on CCOS activities, coverage and staffing, as defined above.

All analyses were adjusted for a linear time trend, seasonality (11 dummy variables for the months February to December), and the 14 time-dependent confounding variables. In addition, analyses of admissions out of hours were adjusted for unit occupancy, and analyses of unit survivors discharged to the ward were adjusted for age, ICNARC physiology score and surgical status.

Interactions between the categorical variables representing CCOS coverage and staffing were tested in the corresponding models.

A sensitivity analysis was conducted for the outcome of CPR before admission by including only those patients in hospital for at least 24 hours before admission, to exclude CPR occurring out of hospital. A sensitivity analysis was also conducted for admissions having all active treatment withdrawn, restricting to active treatment withdrawal occurring within 48 hours of admission, because these may represent futile admissions that are more likely to be averted by a CCOS.

Statistical analyses were performed with Stata 9.2 (StataCorp LP, College Station, TX, USA).

## Results

In all, 130 units were identified both in the CMPD and in survey data. Of these, 111 indicated the presence of CCOS and were contacted to acquire the formal start date; 107 (96%) responded. The four units that did not respond, for which no date for the start of formal outreach services could be identified, were dropped from the analyses. A further 18 units were dropped from the analyses because of missing values in the time-dependent survey data.

Of the original 130 units, 108 (83%) were included in the analyses, of which 79 (73%) had a formal CCOS starting between 1996 and 2004. There was a median of 36.5 (quartiles 24 to 47) months' data after the introduction of CCOS in these units. The 29 units with no formal CCOS or with CCOS starting after 2004 were included as non-intervention sites to improve the modelling of time trends and confounders.

The characteristics of patients in the three subgroups of admissions are described in Table 1.

**Table 1:** Descriptive statistics for all admissions, admissions from the ward and discharges to the ward

Statistics and outcomes	All admissions	Admissions from the ward	Discharges to the ward
Admissions, *n *(percentage)	240,884 (100)	56,082 (23.3)	138,160 (57.4)
Age (years)			
Mean (SD)	59.3 (19.4)	60.1 (19.0)	58.6 (19.7)
Median (quartiles)	64 (48–74)	65 (50–74)	63 (46–74)
Males, *n *(percentage)	139,176 (57.8)	30,437 (54.3)	78,986 (57.2)
ICNARC physiology score			
Mean (SD)	18.2 (10.2)	21.5 (10.8)	14.5 (7.7)
Median (quartiles)	17 (10–24)	20 (14–28)	13 (9–19)
Admission type, *n *(percentage)			
Non-surgical	139,376 (57.9)	56,082 (100)	66,214 (47.9)
Elective surgical	53,563 (22.2)	NA	43,099 (31.2)
Emergency surgical	47,945 (19.9)	NA	28,847 (20.8)
Hospital mortality	235,551 (32.6)	25,847 (46.9)	16,184 (11.7)
Outcomes for admissions from the ward, *n *(percentage)			
CPR 24 hours prior to admission		5,349 (9.6)	
Admission out of hours (22:00–06:59)		16,312 (29.1)	
Active treatment withdrawn		8,670 (15.4)	
Unit mortality		18,040 (32.2)	
Outcomes for discharges to the ward, *n *(percentage)			
Discharge out of hours (22:00–06:59)			8,870 (6.4)
Early discharge due to shortage of beds			5,440 (3.9)
Readmission within 48 hours			1,919 (1.4)

CPR, cardiopulmonary resuscitation; ICNARC, Intensive Care National Audit & Research Centre; NA, not applicable.

**Figure 1:**
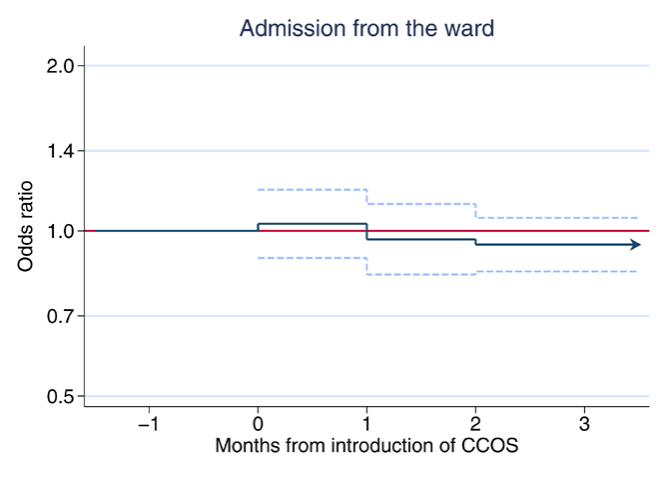
The effect of critical care outreach services (CCOS) for all admissions to the unit. Effect estimate (odds ratio) and 95% confidence interval are shown for the first, second, and third and subsequent months after the introduction of CCOS.

**Figure 2:**
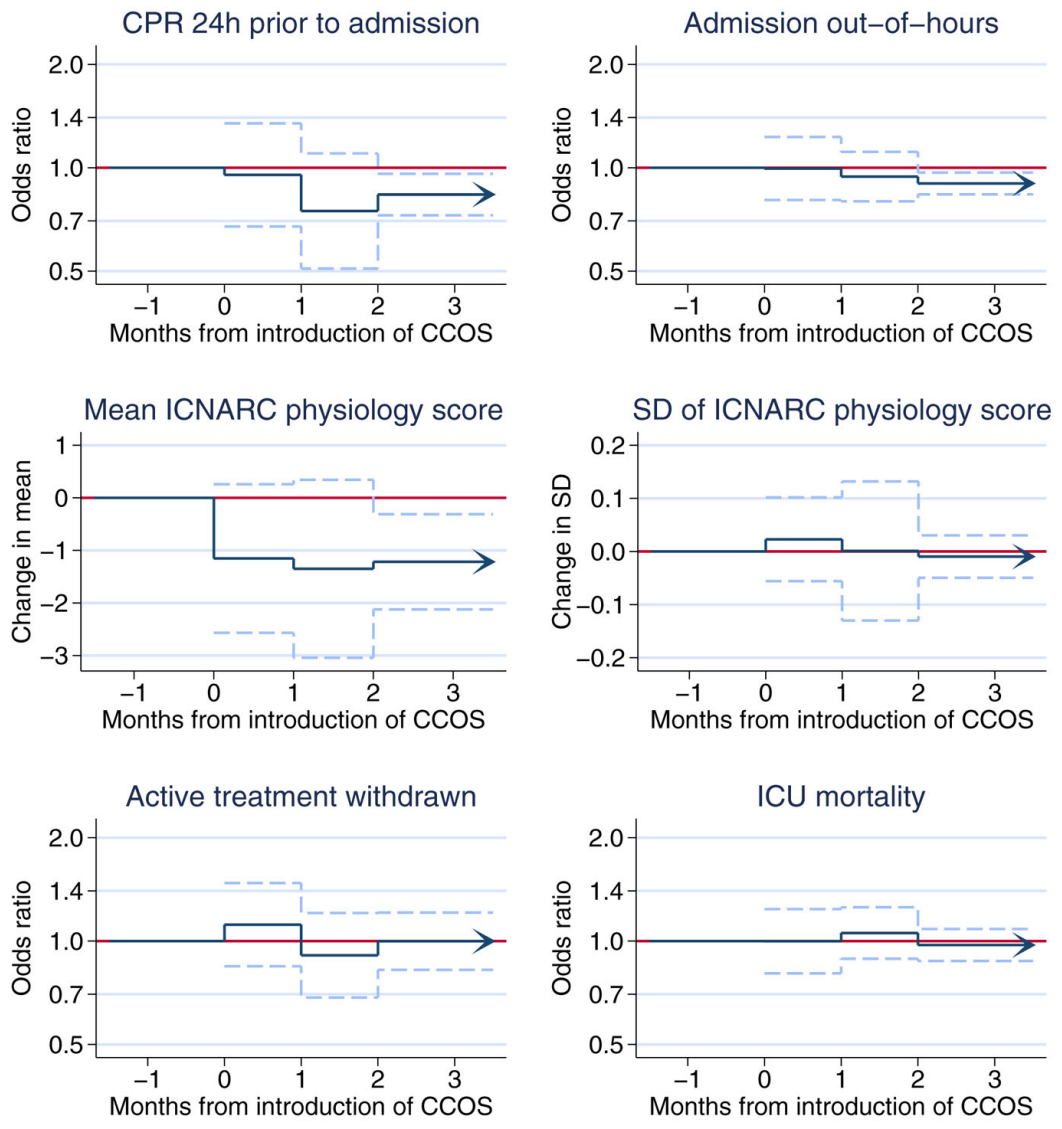
The effect of critical care outreach services (CCOS) for admissions from the ward. Effect estimates and 95% confidence intervals are shown for the first, second, and third and subsequent months after the introduction of CCOS. CPR, cardiopulmonary resuscitation; ICNARC, Intensive Care National Audit & Research Centre; ICU, intensive care unit.

**Figure 3:**
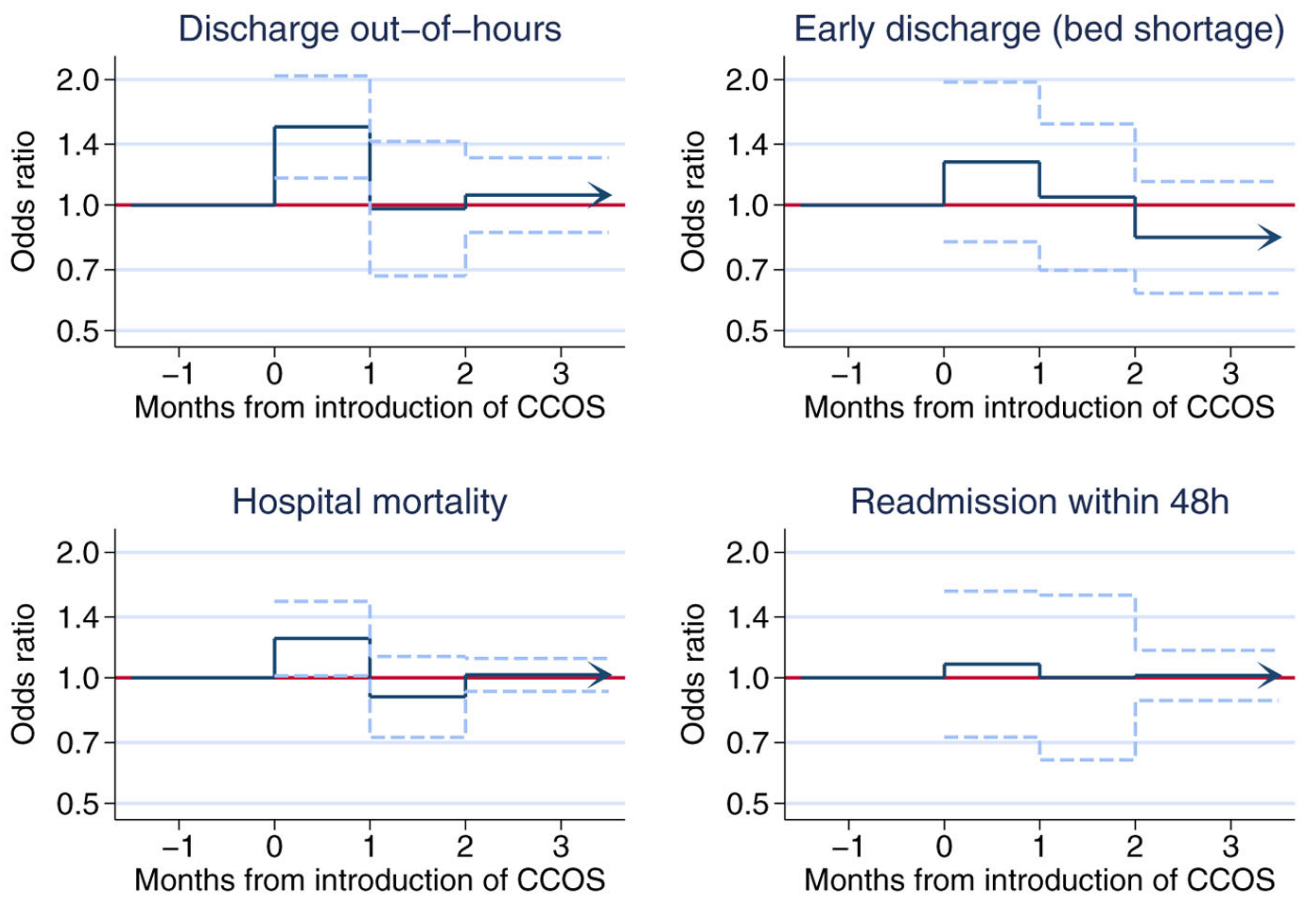
The effect of critical care outreach services (CCOS) for unit survivors discharged to the ward. Effect estimates and 95% confidence intervals are shown for the first, second, and third and subsequent months after the introduction of CCOS.

The effects of the presence of a formal CCOS and its lag over two months on the predefined outcomes for the three subgroups of admissions are shown in Figures 1 to 3. The figures provide a graphical illustration of the effect estimates for the first, second, and third and subsequent months after the introduction of CCOS. The estimates for the first and second months represent the progression from no CCOS to having a CCOS; the estimate for the third and subsequent months represents the sustained effect of CCOS, presumed to remain constant for the life of the CCOS. Full details of the effect estimates are given in Additional file 1. There was no significant change in the proportion of all admissions coming from the ward (Figure 1). For admissions from the ward (Figure 2), the presence of a formal CCOS was associated with a significant decrease in CPR during 24 hours before admission, admission out of hours and the mean ICNARC physiology score. From the third month after the formal start date onwards, the effect estimate (95% confidence interval) and *P *value for these three outcomes were as follows: odds ratio 0.84 (0.73 to 0.96), *P *= 0.012; odds ratio 0.91 (0.84 to 0.97), *P *= 0.012; and decrease in mean 1.22 (0.31 to 2.12), *P *= 0.008, respectively. There was no significant change in the SD of the ICNARC physiology score, the proportion of admissions having all active treatment withdrawn, or unit mortality. For unit survivors discharged to the ward (Figure 3), there was an apparent increase in out-of-hours discharges (and an associated increase in hospital mortality) in the first month after the introduction of CCOS. This effect disappeared in the second and subsequent months.

The sensitivity analyses showed similar results on CPR before admission and active treatment withdrawal in the restricted subgroups.

Full results of the secondary analyses on CCOS activities, coverage and staffing can be found in Additional file 1. We have the following observations.

With regard to CCOS activities, the use of physiological track and trigger warning systems was associated with lower rates of CPR before admission (odds ratio 0.84, 95% confidence interval 0.72 to 0.98, *P *= 0.049) and the SD of the ICNARC physiology score (decrease in SD 0.06 (0.01 to 0.10), *P *= 0.010). Certain other activities were associated with statistically significant changes in outcomes, but with no plausible rationale for causality. For example, the presence of an outpatient follow-up service was associated with characteristics of admissions from the ward. It is likely that these represent spurious findings because of the number of tests performed.

With regard to CCOS coverage, there were some statistically significant differences between coverage categories, but these were not consistent and did not show any expected 'dose-response' pattern.

With regard to CCOS staffing, medical teams were associated with a lower proportion of ward admissions out of hours (odds ratio 0.92 (0.84 to 1.00), *P *= 0.046) and reductions in active treatment withdrawal (odds ratio 0.76 (0.59 to 0.97), *P *= 0.026) in comparison with teams with no medical involvement. Larger teams were associated with a higher proportion of all admissions coming from the ward (odds ratio 1.18 (1.02 to 1.35), *P *= 0.025), increased active treatment withdrawal in admissions from the ward (odds ratio 1.29 (1.02 to 1.64), *P *= 0.033) and higher hospital mortality for patients discharged to the ward (odds ratio 1.11 (1.02 to 1.21), *P *= 0.020) in comparison with smaller teams. The direction of causality in these associations is unclear.

There were no significant interactions between the variables representing CCOS coverage and staffing.

## Discussion

This study found that the presence of a formal CCOS was associated with a significant decrease in CPR rates during 24 hours prior to admission, out-of-hours admission (22:00 to 06:59) and mean ICNARC physiology score for admissions from the ward. There was no evidence for an association between the presence of a formal CCOS and the other outcomes investigated in this study. In particular, there was no effect on unit mortality for patients admitted to the critical care unit from the ward, and no sustained effect was seen on mortality or readmission rates for patients discharged alive from the critical care unit.

Cardiopulmonary arrest is a clinically important adverse event that carries a high mortality. Such an event is often preceded by signs of physiological deterioration [8,9]. The findings in the present study suggest that the use of physiological track and trigger warning systems is an important part of CCOS activity. The use of such a system may lead to earlier intervention when a patient shows signs of deteriorating and may therefore reduce the CPR rate. A wide variety of track and trigger warning systems are in use, with little evidence of reliability, validity or utility [10]. In most previous studies it has been impossible to distinguish any effects of using a track and trigger system from other components of CCOS activity. Only one single centre study has evaluated the effect of introducing a track and trigger system in the absence of a specific CCOS or similar service providing the response [11]. The finding of reduced CPR is consistent with some previous studies in non-randomised before/after comparisons of CCOS or similar services [12-15]. However, other studies, including the MERIT cluster-randomised trial, have reported no significant effects on CPR rates [16-18]. CPR rates in patients admitted to critical care units may be reduced because arrest rates are reduced, but there are also other plausible explanations. It may be that the arrest rate remains the same but resuscitation is attempted less frequently through the more appropriate use of 'do not attempt resuscitation' decisions. Alternatively, it may be that the same number of arrests and resuscitation attempts are still taking place, but fewer of these patients are being admitted to critical care units because the CCOS determine admission to be futile. It is most likely that some combination of all these effects is taking place.

Reductions in out-of-hours admissions to the intensive care unit (ICU) may result from a number of different processes. It may be that patients requiring critical care are being identified early and admitted appropriately during the working day, averting the need to admit the patient as an emergency in the middle of the night. Alternatively, it is possible that in hospitals with a CCOS that does not operate 24 hours per day, at-risk patients identified overnight are being left until the CCOS begins work in the morning rather than being referred directly to the ICU.

The fact that acute severity of illness, as measured by the ICNARC physiology score, was reduced without an associated reduction in mortality may reflect lead-time bias – a reduction in the apparent severity of illness as a result of stabilisation before admission, rather than a true reduction in the underlying severity of illness [19]. However, true severity of illness may be affected by at least three processes if CCOS achieve the stated aim of averting admissions or ensuring timely admission. Averting ICU admissions that can be managed safely on the ward with the assistance of the CCOS would remove some of the least sick patients, resulting in an increase in the average severity of illness. Conversely, averting futile admissions that would not benefit from critical care by the increased used of decisions on treatment limitation would remove some of the sickest patients, resulting in a decrease in the average severity of illness. Finally, ensuring the timely admission of patients requiring critical care may enable them to be admitted at an earlier stage in the disease process, with lower severity of illness.

The fact that other expected changes resulting from CCOS were not evident may be due to a genuine lack of benefit of CCOS or to the variability in the way in which these services were designed and implemented, and the funding available to them, leading to similar variability in their impact. There may be other factors not captured in the survey that could have had an impact on the effects of a CCOS, for example organisational or management and leadership styles or culture. There is some modest impact in places, but we must wait to see whether this will be sustained in the future.

Overall, this study showed a very mixed picture. There is no clear evidence that CCOS have a big impact on patient outcomes. In addition, there do not seem to be any clear characteristics of what should form the optimal CCOS.

The three major strengths of our study are the size, high-quality data and rigorous methodology. We performed a multicentre study on a national scale: data from 108 critical care units were included in the analyses, representing about half of all adult general critical care units in England. The CMPD has been independently evaluated in accordance with criteria for a high-quality database and scored highly [5]. The approach of interrupted time-series analysis has advantages over a simple before/after comparison because it controls for long-term trends and seasonality in the data, but it may be influenced by other events occurring at about the same time as the event of interest (historical bias) [20,21]. In the CMPD, we have time-series data for many critical care units (namely panel data or cross-sectional time-series data) [22]. The introduction of outreach services at different times in different locations produces a natural experiment by which we can reduce the effects of historical bias. Population-averaged panel-data models estimate the consistent (average) effect of CCOS across hospitals. This effect estimate is of most relevance for policy and planning decisions. All major potential confounding factors were identified and included in the study.

There were several limitations to our study. First, the variations in the way in which CCOS have been implemented decrease our ability to analyse and understand their impact. However, because CCOS are widespread in England [3], a randomised controlled trial of their effectiveness is now infeasible. Well-controlled, multicentre observational studies are therefore likely to be the best way to gain additional insight into this topic. Second, the delivery of CCOS may have changed over the course of the study period. We were limited to information on the set-up of CCOS obtained from a survey conducted at a single point in time; however, had more detailed data been available, it is doubtful whether it would have been possible to fit such a complex model. Third, we observed associations with the introduction of CCOS but are unable to attribute causality. For example, we cannot determine from the data whether the decrease in CPR before admission to ICU was due to the prevention of arrests by earlier referral or to an increase in decisions on treatment limitation. Bradford Hill [23] has identified nine 'considerations for causality': strength of the association; consistency across observers, places, circumstances and times; specificity (that is, that the same association is not observed in other settings); temporal relationship; biological gradient (that is, dose-response); plausibility; coherence with what is already known in the area; experiment (which provides the strongest argument, when available); and analogy with similar situations. The multicentre interrupted time-series approach helps to establish consistency, specificity and temporal relationships. However, none of the associations could be considered to be overwhelmingly strong, and certain results, particularly among the secondary analyses, failed on consideration of plausibility or biological gradient. Fourth, although the population-averaged effect is the most relevant for policy decisions, it does not measure the expected benefit for an individual patient, because the population includes individuals with no potential to gain from the presence of CCOS. For this reason, we concentrated the analyses on subpopulations with the most potential to benefit. Finally, length of stay in critical care and in hospital may be important performance indicators and are strongly associated with costs, but these were not investigated because they are highly skewed variables, making it difficult to identify significant population-averaged effects.

Further large, multicentre, prospective studies are required to identify which aspects of CCOS are truly effective. We propose to evaluate the impact of outreach services, at the patient level, by prospectively identifying admissions in the CMPD receiving outreach before and/or after their critical care episode.

## Conclusion

Although some effects of CCOS were found, there is no clear evidence that CCOS have a big impact on outcomes of patients admitted to critical care. No clear characteristics of what should form the optimal CCOS could be identified, except that the use of physiological track and trigger warning systems seems potentially beneficial. There is some modest impact in places, but we must wait to see whether this will be sustained in the future and whether this is associated with improvements in important patient outcomes. Further large, multicentre prospective studies are required.

## Key messages

• CCOS have been widely introduced in England with the aims of averting admissions to critical care, ensuring timely admission, enabling discharge and educating the ward staff.

• Our interrupted time-series analysis demonstrates reductions in the proportion of admissions receiving CPR before admission, admission out of hours, and severity of illness for patients admitted to the ICU from the ward, but no effect on unit mortality.

• There were no sustained effects on outcomes for unit survivors discharged to the ward.

• Analysis of specific CCOS activities suggested that changes in admission characteristics may be attributable in part to the use of physiological track and trigger warning systems.

## Abbreviations

CCOS = critical care outreach services; CMPD = Case Mix Programme Database; CPR = cardiopulmonary resuscitation; ICNARC = Intensive Care National Audit & Research Centre; ICU = intensive care unit; NHS = National Health Service.

## Competing interests

The authors declare that they have no competing interests.

## Authors' contributions

HG and DAH led the design and analysis of the study and drafted the manuscript. GJP, KD, CPS and KR contributed to the design of the study, interpretation of results, and critical revision of the manuscript. All authors read and approved the final manuscript.

## Supplementary Material

Additional file 1 A PDF file containing five tables listing detailed results of all primary and secondary analyses and sensitivity analyses. Click here for file
