# Supplementary material for: The impact of the introduction of critical care outreach services in England: a multicentre interrupted time-series analysis
Source: Crit Care. 2007 Sep 18;11(5):R113. doi: 10.1186/cc6163 (PMC2556766; doi:10.1186/cc6163)
Supplement: Additional file 1 — A PDF file containing five tables listing detailed results of all primary and secondary analyses and sensitivity analyses. [file cc6163-S1.pdf]

Table S1 Effects of formal start of critical care outreach services (CCOS) on outcomes

Primary exposure variable	Effect estimate (95% CI) compared with no CCOS	P-value
Model1a, Admission from ward	*Odds ratio*	0.687[a]
CCOS month 1	1.03 (0.89, 1.19)	0.682
CCOS month 2	0.97 (0.83, 1.12)	0.641
CCOS month 3 and following	0.94 (0.84, 1.06)	0.316
Model2a, CPR 24 hours prior to admission	*Odds ratio*	0.049[a]
CCOS month 1	0.95 (0.67, 1.35)	0.787
CCOS month 2	0.75 (0.51, 1.10)	0.142
CCOS month 3 and following	0.84 (0.73, 0.96)	0.012
Model2b, Admission out-of-hours	*Odds ratio*	0.039[a]
CCOS month 1	1.00 (0.81, 1.24)	0.996
CCOS month 2	0.95 (0.81, 1.12)	0.534
CCOS month 3 and following	0.91 (0.84, 0.97)	0.005
Model2c, ICNARC physiology score	*Change in mean*	0.049[a]
CCOS month 1	−1.15 (−2.57, 0.26)	0.109
CCOS month 2	−1.35 (−3.04, 0.34)	0.118
CCOS month 3 and following	−1.22 (−2.12, −0.31)	0.008
Model2c, ICNARC physiology score	*Change in SD*	0.870[a]
CCOS month 1	0.023 (−0.056, 0.102)	0.568
CCOS month 2	0.001 (−0.130, 0.132)	0.988
CCOS month 3 and following	−0.010 (−0.050, 0.030)	0.638
Model2d, Active treatment withdrawn	*Odds ratio*	0.739[a]
CCOS month 1	1.12 (0.84, 1.48)	0.440
CCOS month 2	0.91 (0.68, 1.21)	0.510
CCOS month 3 and following	1.00 (0.82, 1.21)	0.992
Model2e, Mortality in ICU	*Odds ratio*	0.757[a]
CCOS month 1	1.00 (0.81, 1.24)	0.993
CCOS month 2	1.06 (0.89, 1.25)	0.537
CCOS month 3 and following	0.97 (0.87, 1.08)	0.628
Model3a, Discharge out-of-hours	*Odds ratio*	0.003[a]
CCOS month 1	1.57 (1.19, 2.07)	0.001
CCOS month 2	1.01 (0.69, 1.46)	0.977
CCOS month 3 and following	1.08 (0.89, 1.31)	0.453
Model3b, Early discharge due to shortage of beds	*Odds ratio*	0.310[a]
CCOS month 1	1.30 (0.84, 2.01)	0.242
CCOS month 2	1.08 (0.72, 1.61)	0.708
CCOS month 3 and following	0.86 (0.64, 1.15)	0.304
Model3c, Hospital mortality	*Odds ratio*	0.069[a]
CCOS month 1	1.28 (1.05, 1.57)	0.016
CCOS month 2	0.94 (0.76, 1.17)	0.578
CCOS month 3 and following	1.05 (0.96, 1.15)	0.277
Model3d, Readmission within 48 hours of discharge	*Odds ratio*	0.944[a]
CCOS month 1	1.09 (0.73, 1.64)	0.676
CCOS month 2	1.02 (0.64, 1.62)	0.932
CCOS month 3 and following	1.03 (0.90, 1.19)	0.649

[a]P-value for overall effect of CCOS. CI, confidence interval; CPR, cardiopulmonary resuscitation; ICNARC, Intensive Care National Audit & Research Centre; ICU, intensive care unit; SD, standard deviation.

Table S2 Effects of critical care outreach service (CCOS) activities on outcomes

Secondary exposure variable	Effect estimate (95% CI) compared with no CCOS	P-value
Model1a, Admission from ward	*Odds ratio*	0.603 [a]
Ward follow-up	0.89 (0.75, 1.06)	0.190
Outpatient follow-up	1.12 (0.99, 1.28)	0.073
Telephone advice	1.01 (0.87, 1.18)	0.859
Direct bedside clinical support	0.97 (0.75, 1.25)	0.815
Informal bedside teaching	1.07 (0.84, 1.37)	0.563
Formal educational courses	0.96 (0.86, 1.07)	0.466
Use of early warning/track & trigger	1.07 (0.93, 1.22)	0.338
Audit & evaluation of outreach activity	1.04 (0.89, 1.21)	0.599
Model2a, CPR 24 hours prior to admission	*Odds ratio*	0.111 [a]
Ward follow-up	1.24 (0.97, 1.57)	0.081
Outpatient follow-up	1.00 (0.85, 1.19)	0.977
Telephone advice	0.99 (0.85, 1.15)	0.911
Direct bedside clinical support	0.90 (0.67, 1.21)	0.500
Informal bedside teaching	1.00 (0.65, 1.54)	0.994
Formal educational courses	0.89 (0.73, 1.07)	0.205
Use of early warning/track & trigger	0.84 (0.72, 0.98)	0.029
Audit & evaluation of outreach activity	0.92 (0.76, 1.10)	0.346
Model2b, Admission out-of-hours	*Odds ratio*	0.152 [a]
Ward follow-up	0.97 (0.89, 1.07)	0.586
Outpatient follow-up	0.99 (0.92, 1.06)	0.734
Telephone advice	1.08 (1.01, 1.15)	0.017
Direct bedside clinical support	0.97 (0.88, 1.07)	0.565
Informal bedside teaching	1.05 (0.96, 1.15)	0.268
Formal educational courses	0.97 (0.90, 1.04)	0.363
Use of early warning/track & trigger	1.01 (0.93, 1.10)	0.746
Audit & evaluation of outreach activity	0.92 (0.82, 1.02)	0.096
Model2c, ICNARC physiology score	*Change in mean*	0.402 [a]
Ward follow-up	0.99 (−0.57, 2.55)	0.213
Outpatient follow-up	0.33 (−0.67, 1.33)	0.518
Telephone advice	−0.13 (−1.04, 0.77)	0.772
Direct bedside clinical support	−1.00 (−3.29, 1.28)	0.387
Informal bedside teaching	0.20 (−2.08, 2.48)	0.863
Formal educational courses	0.10 (−0.96, 1.16)	0.854
Use of early warning/track & trigger	−0.83 (−1.91, 0.25)	0.130
Audit & evaluation of outreach activity	−0.13 (−1.28, 1.02)	0.824
Model2c, ICNARC physiology score	*Change in SD*	0.098 [a]
Ward follow-up	−0.042 (−0.093, 0.010)	0.111
Outpatient follow-up	−0.011 (−0.044, 0.023)	0.536
Telephone advice	0.014 (−0.021, 0.048)	0.442
Direct bedside clinical support	0.017 (−0.030, 0.063)	0.487
Informal bedside teaching	0.037 (−0.022, 0.095)	0.220
Formal educational courses	0.022 (−0.016, 0.059)	0.261
Use of early warning/track & trigger	−0.058 (−0.103, −0.014)	0.010
Audit & evaluation of outreach activity	0.011 (−0.049, 0.071)	0.716
Model2d, Active treatment withdrawn	*Odds ratio*	0.006 [a]
Ward follow-up	1.04 (0.83, 1.31)	0.740
Outpatient follow-up	1.27 (1.09, 1.47)	0.002
Telephone advice	0.80 (0.67, 0.97)	0.021
Direct bedside clinical support	1.03 (0.79, 1.35)	0.815
Informal bedside teaching	0.92 (0.65, 1.31)	0.653
Formal educational courses	1.15 (0.93, 1.41)	0.201
Use of early warning/track & trigger	0.86 (0.67, 1.11)	0.249
Audit & evaluation of outreach activity	1.15 (0.89, 1.47)	0.281

Model2e, Mortality in ICU	*Odds ratio*	0.655 [a]
Ward follow-up	1.01 (0.89, 1.16)	0.838
Outpatient follow-up	0.92 (0.83, 1.01)	0.087
Telephone advice	0.96 (0.87, 1.06)	0.448
Direct bedside clinical support	0.97 (0.85, 1.10)	0.640
Informal bedside teaching	1.03 (0.90, 1.18)	0.706
Formal educational courses	0.96 (0.86, 1.07)	0.468
Use of early warning/track & trigger	0.99 (0.89, 1.11)	0.905
Audit & evaluation of outreach activity	1.01 (0.88, 1.16)	0.855
Model3a, Discharge out-of-hours	*Odds ratio*	0.027 [a]
Ward follow-up	0.95 (0.76, 1.20)	0.688
Outpatient follow-up	1.10 (0.92, 1.31)	0.309
Telephone advice	1.13 (0.95, 1.35)	0.160
Direct bedside clinical support	0.72 (0.55, 0.94)	0.014
Informal bedside teaching	1.23 (0.99, 1.52)	0.061
Formal educational courses	1.00 (0.83, 1.20)	0.994
Use of early warning/track & trigger	1.02 (0.72, 1.44)	0.906
Audit & evaluation of outreach activity	1.23 (1.01, 1.50)	0.038
Model3b, Early discharge due to shortage of beds	*Odds ratio*	0.710 [a]
Ward follow-up	0.91 (0.63, 1.30)	0.598
Outpatient follow-up	0.88 (0.66, 1.17)	0.380
Telephone advice	1.04 (0.79, 1.36)	0.785
Direct bedside clinical support	1.05 (0.70, 1.57)	0.805
Informal bedside teaching	0.97 (0.71, 1.33)	0.870
Formal educational courses	0.95 (0.73, 1.26)	0.740
Use of early warning/track & trigger	1.29 (0.93, 1.80)	0.132
Audit & evaluation of outreach activity	0.84 (0.56, 1.26)	0.391
Model3c, Hospital deaths	*Odds ratio*	0.114 [a]
Ward follow-up	0.98 (0.82, 1.18)	0.862
Outpatient follow-up	0.94 (0.85, 1.03)	0.193
Telephone advice	1.09 (0.96, 1.23)	0.169
Direct bedside clinical support	0.79 (0.64, 0.96)	0.020
Informal bedside teaching	1.15 (1.01, 1.32)	0.037
Formal educational courses	1.03 (0.94, 1.12)	0.530
Use of early warning/track & trigger	1.05 (0.94, 1.16)	0.409
Audit & evaluation of outreach activity	1.07 (0.92, 1.23)	0.381
Model3d, Readmission within 48 hours of discharge	*Odds ratio*	0.411 [a]
Ward follow-up	1.00 (0.80, 1.25)	0.972
Outpatient follow-up	0.98 (0.86, 1.11)	0.739
Telephone advice	1.01 (0.84, 1.21)	0.904
Direct bedside clinical support	0.97 (0.76, 1.23)	0.781
Informal bedside teaching	1.17 (0.96, 1.42)	0.123
Formal educational courses	1.00 (0.86, 1.15)	0.950
Use of early warning/track & trigger	0.88 (0.70, 1.09)	0.247
Audit & evaluation of outreach activity	1.08 (0.83, 1.40)	0.564

[a]P-value for overall effect of CCOS activities. CI, confidence interval; CPR, cardiopulmonary resuscitation; ICNARC, Intensive Care National Audit & Research Centre; ICU, intensive care unit; SD, standard deviation.

Table S3 Effects of the coverage of critical care outreach services (CCOS) on outcomes

Secondary exposure variable	Effect estimate (95% CI) compared with no CCOS	P-value
Model1a, Admission from ward	*Odds ratio*	0.438[a]/0.330[b]
24 hours 7 days a week, 100% ward coverage	0.93 (0.77, 1.12)	0.423
12-23 hours 7 days, 100% ward coverage	1.20 (0.87, 1.65)	0.260
< 12 hours 7 days, 100% ward coverage	0.95 (0.79, 1.13)	0.549
Selected days, 100% ward coverage	0.88 (0.76, 1.01)	0.068
24 hours 7 days, <100% ward coverage	0.93 (0.75, 1.15)	0.496
12-23 hours 7 days, <100% ward coverage	1.20 (0.87, 1.67)	0.272
< 12 hours 7 days, <100% ward coverage	0.95 (0.80, 1.13)	0.556
Selected days, <100% ward coverage	0.88 (0.76, 1.02)	0.092
Model2a, CPR 24 hours prior to admission	*Odds ratio*	0.215[a]/0.015[b]
24 hours 7 days a week, 100% ward coverage	0.67 (0.50, 0.89)	0.005
12-23 hours 7 days, 100% ward coverage	0.96 (0.66, 1.40)	0.838
< 12 hours 7 days, 100% ward coverage	0.84 (0.68, 1.04)	0.105
Selected days, 100% ward coverage	0.76 (0.63, 0.91)	0.004
24 hours 7 days, <100% ward coverage	0.80 (0.61, 1.05)	0.103
12-23 hours 7 days, <100% ward coverage	1.15 (0.75, 1.74)	0.526
< 12 hours 7 days, <100% ward coverage	1.00 (0.80, 1.25)	0.979
Selected days, <100% ward coverage	0.90 (0.73, 1.11)	0.329
Model2b, Admission out-of-hours	*Odds ratio*	0.456[a] /0.085[b]
24 hours 7 days a week, 100% ward coverage	0.95 (0.87, 1.03)	0.220
12-23 hours 7 days, 100% ward coverage	0.97 (0.85, 1.10)	0.581
< 12 hours 7 days, 100% ward coverage	0.90 (0.81, 1.00)	0.055
Selected days, 100% ward coverage	0.89 (0.79, 1.00)	0.045
24 hours 7 days, <100% ward coverage	0.93 (0.83, 1.03)	0.171
12-23 hours 7 days, <100% ward coverage	0.94 (0.81, 1.10)	0.455
< 12 hours 7 days, <100% ward coverage	0.88 (0.80, 0.98)	0.015
Selected days, <100% ward coverage	0.87 (0.77, 0.97)	0.016
Model2c, ICNARC physiology score	*Change in mean*	0.013[a] /0.001[b]
24 hours 7 days a week, 100% ward coverage	−1.49 (−3.12, 0.14)	0.072
12-23 hours 7 days, 100% ward coverage	0.38 (−1.17, 1.94)	0.629
< 12 hours 7 days, 100% ward coverage	−1.19 (−2.42, 0.04)	0.058
Selected days, 100% ward coverage	−2.51 (−3.72, −1.30)	< 0.001
24 hours 7 days, <100% ward coverage	−0.43 (−2.12, 1.26)	0.621
12-23 hours 7 days, <100% ward coverage	1.45 (−0.40, 3.29)	0.124
< 12 hours 7 days, <100% ward coverage	−0.12 (−1.62, 1.38)	0.872
Selected days, <100% ward coverage	−1.44 (−2.62, −0.27)	0.016
Model2c, SD of ICNARC physiology score	*Change in SD*	0.434[a] /0.565[b]
24 hours 7 days a week, 100% ward coverage	−0.036 (−0.101, 0.030)	0.285
12-23 hours 7 days, 100% ward coverage	−0.006 (−0.074, 0.062)	0.865
< 12 hours 7 days, 100% ward coverage	−0.034 (−0.089, 0.021)	0.221
Selected days, 100% ward coverage	−0.004 (−0.061, 0.052)	0.882
24 hours 7 days, <100% ward coverage	−0.004 (−0.078, 0.070)	0.917
12-23 hours 7 days, <100% ward coverage	0.026 (−0.057, 0.108)	0.540
< 12 hours 7 days, <100% ward coverage	−0.003 (−0.068, 0.063)	0.939
Selected days, <100% ward coverage	0.027 (−0.026, 0.080)	0.310
Model2d, Active treatment withdrawn	*Odds ratio*	0.004[a] /0.008[b]
24 hours 7 days a week, 100% ward coverage	1.10 (0.84, 1.46)	0.479
12-23 hours 7 days, 100% ward coverage	1.52 (1.13, 2.05)	0.006
< 12 hours 7 days, 100% ward coverage	1.12 (0.88, 1.42)	0.373
Selected days, 100% ward coverage	0.79 (0.61, 1.03)	0.083
24 hours 7 days, <100% ward coverage	1.00 (0.76, 1.31)	0.985
12-23 hours 7 days, <100% ward coverage	1.37 (0.94, 2.01)	0.102
< 12 hours 7 days, <100% ward coverage	1.01 (0.75, 1.35)	0.956
Selected days, <100% ward coverage	0.72 (0.53, 0.96)	0.027

Model2e, Mortality in ICU	*Odds ratio*	0.860[a] /0.924[b]
24 hours 7 days a week, 100% ward coverage	0.98 (0.83, 1.16)	0.805
12-23 hours 7 days, 100% ward coverage	0.98 (0.86, 1.12)	0.821
< 12 hours 7 days, 100% ward coverage	1.04 (0.88, 1.22)	0.653
Selected days, 100% ward coverage	0.93 (0.79, 1.10)	0.401
24 hours 7 days, <100% ward coverage	0.99 (0.82, 1.18)	0.873
12-23 hours 7 days, <100% ward coverage	0.99 (0.82, 1.20)	0.927
< 12 hours 7 days, <100% ward coverage	1.04 (0.88, 1.24)	0.631
Selected days, <100% ward coverage	0.94 (0.79, 1.12)	0.466
Model3a, Discharge out-of-hours	*Odds ratio*	0.683[a] /0.733[b]
24 hours 7 days a week, 100% ward coverage	1.05 (0.85, 1.29)	0.640
12-23 hours 7 days, 100% ward coverage	1.42 (0.86, 2.37)	0.174
< 12 hours 7 days, 100% ward coverage	0.98 (0.77, 1.23)	0.840
Selected days, 100% ward coverage	1.09 (0.87, 1.36)	0.466
24 hours 7 days, <100% ward coverage	1.03 (0.81, 1.29)	0.824
12-23 hours 7 days, <100% ward coverage	1.39 (0.82, 2.36)	0.222
< 12 hours 7 days, <100% ward coverage	0.95 (0.71, 1.28)	0.751
Selected days, <100% ward coverage	1.06 (0.86, 1.31)	0.572
Model3b, Early discharge due to shortage of beds	*Odds ratio*	0.037[a] /0.066[b]
24 hours 7 days a week, 100% ward coverage	0.58 (0.27, 1.24)	0.159
12-23 hours 7 days, 100% ward coverage	0.63 (0.37, 1.09)	0.099
< 12 hours 7 days, 100% ward coverage	0.77 (0.54, 1.11)	0.164
Selected days, 100% ward coverage	1.22 (0.88, 1.68)	0.236
24 hours 7 days, <100% ward coverage	0.60 (0.30, 1.20)	0.147
12-23 hours 7 days, <100% ward coverage	0.65 (0.35, 1.19)	0.163
< 12 hours 7 days, <100% ward coverage	0.80 (0.52, 1.22)	0.295
Selected days, <100% ward coverage	1.25 (0.83, 1.88)	0.278
Model3c, Hospital deaths	*Odds ratio*	0.089[a] /0.086[b]
24 hours 7 days a week, 100% ward coverage	1.09 (0.94, 1.28)	0.259
12-23 hours 7 days, 100% ward coverage	1.16 (1.04, 1.30)	0.009
< 12 hours 7 days, 100% ward coverage	1.00 (0.87, 1.15)	0.991
Selected days, 100% ward coverage	1.03 (0.92, 1.15)	0.613
24 hours 7 days, <100% ward coverage	1.10 (0.97, 1.26)	0.131
12-23 hours 7 days, <100% ward coverage	1.17 (1.02, 1.35)	0.025
< 12 hours 7 days, <100% ward coverage	1.01 (0.90, 1.14)	0.872
Selected days, <100% ward coverage	1.04 (0.94, 1.15)	0.434
Model3d, Readmission within 48 hours of discharge	*Odds ratio*	0.257[a] /0.341[b]
24 hours 7 days a week, 100% ward coverage	1.02 (0.74, 1.40)	0.927
12-23 hours 7 days, 100% ward coverage	1.13 (0.95, 1.34)	0.154
< 12 hours 7 days, 100% ward coverage	0.93 (0.78, 1.11)	0.411
Selected days, 100% ward coverage	1.10 (0.92, 1.31)	0.305
24 hours 7 days, <100% ward coverage	0.99 (0.72, 1.36)	0.941
12-23 hours 7 days, <100% ward coverage	1.10 (0.89, 1.37)	0.378
< 12 hours 7 days, <100% ward coverage	0.90 (0.75, 1.10)	0.306
Selected days, <100% ward coverage	1.07 (0.88, 1.30)	0.507

[a]P-value for difference among the secondary exposure categories. [b]P-value for overall effect of CCOS. CI, confidence interval; CPR, cardiopulmonary resuscitation; ICNARC, Intensive Care National Audit & Research Centre; ICU, intensive care unit; SD, standard deviation.

Table S4 Effects of the staffing of critical care outreach services (CCOS) on outcomes

Secondary exposure variable	Effect estimate (95% CI) compared with no CCOS	P-value
Model1a, Admission from ward	*Odds ratio*	0.079[a]/0.070[b]
Medical team	1.07 (0.88, 1.30)	0.500
Large team	1.18 (1.02, 1.35)	0.025
Model2a, CPR 24 hours prior to admission	*Odds ratio*	0.374[a] /0.029[b]
Medical team	0.87 (0.71, 1.08)	0.222
Large team	0.93 (0.77, 1.13)	0.485
Model2b, Admission out-of-hours	*Odds ratio*	0.090[a] /0.003[b]
Medical team	0.92 (0.84, 1.00)	0.046
Large team	0.99 (0.91, 1.07)	0.809
Model2c, ICNARC physiology score	*Change in mean*	1.000[a] /0.054[b]
Medical team	0.02 (−1.36, 1.40)	0.978
Large team	0.01 (−1.12, 1.14)	0.988
Model2c, ICNARC physiology score	*Change in SD*	0.360[a] /0.502[b]
Medical team	−0.006 (−0.072, 0.060)	0.862
Large team	−0.033 (−0.079, 0.013)	0.157
Model2d, Active treatment withdrawn	*Odds ratio*	0.006[a] /0.015[b]
Medical team	0.76 (0.59, 0.97)	0.026
Large team	1.29 (1.02, 1.64)	0.033
Model2e, Mortality in ICU	*Odds ratio*	0.276[a] /0.390[b]
Medical team	0.91 (0.81, 1.03)	0.123
Large team	0.99 (0.88, 1.10)	0.812
Model3a, Discharge out-of-hours	*Odds ratio*	0.297[a] /0.342[b]
Medical team	0.84 (0.65, 1.09)	0.192
Large team	1.02 (0.76, 1.36)	0.909
Model3b, Early discharge due to shortage of beds	*Odds ratio*	0.153[a] /0.249[b]
Medical team	0.84 (0.55, 1.27)	0.404
Large team	0.78 (0.54, 1.12)	0.177
Model3c, Hospital deaths	*Odds ratio*	0.062[a] /0.051[b]
Medical team	1.01 (0.93, 1.10)	0.801
Large team	1.11 (1.02, 1.21)	0.020
Model3d, Readmission within 48 hours of discharge	*Odds ratio*	0.634[a] /0.750[b]
Medical team	1.06 (0.87, 1.30)	0.535
Large team	1.05 (0.89, 1.24)	0.587

[a]P-value for difference among the secondary exposure categories. [b]P-value for overall effect of CCOS. CI, confidence interval; CPR, cardiopulmonary resuscitation; ICNARC, Intensive Care National Audit & Research Centre; ICU, intensive care unit; SD, standard deviation.

Table S5 Results of sensitivity analyses

Primary exposure variable	Effect estimate (95% CI) compared with no CCOS	P-value
Model2a, CPR 24 hours prior to admission[b]	*Odds ratio*	0.086[a]
CCOS month 1	0.81 (0.51, 1.29)	0.371
CCOS month 2	0.92 (0.57, 1.49)	0.736
CCOS month 3 and following	0.82 (0.70, 0.96)	0.015
Model2d, Active treatment withdrawn[c]	*Odds ratio*	0.965[a]
CCOS month 1	0.92 (0.55, 1.52)	0.740
CCOS month 2	0.92 (0.62, 1.38)	0.695
CCOS month 3 and following	0.97 (0.77, 1.22)	0.782

[a]P-value for overall effect of CCOS. [b]Restricted to admissions in hospital for at least 24 hours prior to ICU admission. [c]Restricted to active treatment withdrawal occurring within 48 hours of ICU admission. CCOS, critical care outreach service; CI, confidence interval; CPR, cardiopulmonary resuscitation; ICU, intensive care unit.
